# Evaluation of the true precocious puberty rats induced by neonatal administration of Danazol: Therapeutic effects of nourishing "Yin"- removing "Fire" Chinese herb mixture

**DOI:** 10.1186/1477-7827-3-38

**Published:** 2005-08-22

**Authors:** Zhanzhuang Tian, Hong Zhao, Yan Sun, Depei Cai, Boying Chen

**Affiliations:** 1 Department of Neurobiology and Integrative Medicine, Shanghai Medical College of Fudan University (Formerly Shanghai Medical University), P.O. Box 291, 138 Yi-Xue-Yuan Road, 200032 Shanghai, P. R. China; 2 Children's Hospital of Fudan University, 183 Feng-Lin Road, 200032 Shanghai, P. R. China

## Abstract

**Background:**

Nourishing "Yin"-Removing "Fire" Chinese Herb Mixture, a traditional herb-based formulation, has been successfully used for the management of idiopathic true precocious puberty (IPP) for more than thirty years. Precocious puberty rat model by neonatal administration of Danazol was used to investigate the effects of the herb mixture on the advanced sexual development of the rats, and the expression of hypothalamic gonadotropin-releasing hormone (GnRH), which is the important regulator for the hypothalamus-pituitary-gonadal axis, particularly at puberty.

**Methods:**

Female Sprague-Dawley rats were divided into five groups: intact normal (N), IPP model (M), vehicle with no IPP (V), IPP model exposed to herb mixture (HM) and IPP model exposed to saline (S). Rats at 5 days of age were given a single subcutaneous injection of 300 microgram of Danazol dissolved in 25 microliter vehicle of propylene glycol-ethanol (1:1, v/v), to establish the precocious puberty model. From the day 15, rats in HM and S groups were continuously fed with either Nourishing "Yin"-Removing "Fire" Chinese Herb Mixture 2 ml or saline 2 ml, until 3 consecutive regular estrous cycles were established. The day of vaginal opening and the day of setup regular estrous cycle of the rats were observed. Blood concentration of estrogen was determined by radioimmunoassay. Immunohistochemistry and RT-PCR analysis were used to explore the expression of GnRH.

**Results:**

The day of vaginal opening and first estrous showed significant advancement in M compared with N and V (p < 0.05, respectively). The blood estrogen level increased significantly in M compared with those in other groups (about 28 days of age, at the time of vaginal opening in M rats) (p < 0.05, respectively). GnRH cells in rostral medial septum (MS), Broca diagonal band nucleus (DBB) and the medial preoptic area (MPOA), were calculated. The number in M was less than those in N and V (p < 0.05, respectively). The number was significantly higher in HM than that in M (p < 0.05). The GnRH mRNA expression increased significantly in M compared with that in N and V (p < 0.05).

**Conclusion:**

The true precocious puberty model by neonatal administration of Danazol in female rats showed augmented expression of hypothalamic GnRH; the Nourishing "Yin"-Removing "Fire" Chinese Herb Mixture down-regulated the increased GnRH expression, and significantly delayed the sexual development of the precocious puberty rat.

## Background

Sexual precocity is one of the most popular endocrine disorders in children, incidence of which is about 0.6% throughout the world [1]. It is 10 times more common in girls than in boys of the disease. Precocious puberty is the appearance of the secondary sexual characteristics before age 8 years and can be mainly classified into true precocious puberty (GnRH-dependent sexual precocity) and incomplete isosexual precocity (GnRH-independent sexual precocity). The term of true precocious puberty properly applies only to sexual precocity mediated by premature activation of the hypothalamic-pituitary-ovarian axis usually before 8 year-old. Though central nervous system tumors such as hamartomas and astrocytomas may cause true precocious puberty, most cases have no organic disease, which is defined idiopathic true precocious puberty (IPP) [2].

Though experimental models of precocious puberty have been induced in female rats by neonatal injection of testosterone, estradiol or melatonin, these rats developed persistent vaginal estrous or disturbance of cyclicity (predominance of estrous smear) shortly after the day of first estrous. It has been reported that the neonatal administration of Danazol may affect the hypothalamic pituitary axis with the rapid rate of maturation, which may serve as a model for analyzing true precocious puberty [3].

Several medications have been reported to be effective against true precocious puberty, which include the GnRH analogues [4], progesterone prescriptions and Chinese herbal medicine (CHM). Nourishing "Yin"-Removing "Fire" Chinese Herb Mixture, a traditional herb-based formulation, has been successfully used for the management of IPP by us for more than thirty years. It has been clinically verified to successfully modulate the course of pubertal development and optimize skeletal development in children with precocious puberty, but without notable side effects [5]. The present study was to observe the effects of the herb mixture on the true precocious rat model.

## Methods

### Animals

Female Sprague-Dawley rats at 3 days of age in company with the maters were purchased from Medical Experimental Animals Center of Fudan University (Shanghai, China). Animals were housed under laminar flow in an isolated room with controlled temperature and at a 12 /12 (light /dark) schedule. The model [3] litters at day 5 (the day of birth was termed day 1) were given a single subcutaneous injection of 300 μg of Danazol (Hualian Pharm Ltd, Shanghai, China) dissolved in 25 μl vehicle of propylene glycol-ethanol (1:1, v/v), and allowed to grow without further treatment. The animals were weaned on day 23, and were examined daily for vaginal opening afterwards, and then the daily vaginal smears were examined. All experimental procedures involving the use of animals were conducted in accordance with NIH Guidelines and were reviewed and approved by the Animal Use and Care Committee for the Fudan University.

In the Experiment 1, rats were used to observe the day of vaginal opening and the day of setup regular oestrous cycle, which were divided into five groups: intact normal (N), IPP model (M), vehicle with no IPP (V), IPP model exposed to herb mixture (HM) and IPP model exposed to saline (S). Daily vaginal smears were checked until 3 consecutive regular 4 or 5 days estrous cycles were established. The day of diestrous I of the first cycle in the 3 consecutive regular estrous cycles was determined as the day of setup regular estrous cycle. From the day 15, rats in HM and S groups were continuously fed with either Nourishing "Yin"-Removing "Fire" Chinese Herb Mixture 2 ml or saline 2 ml, until 3 consecutive regular estrous cycles were established.

### RIA of blood estrogen concentration

At the time of vaginal opening in model rats, the blood samples of all the rats were collected from tail veins respectively. The plasma was separated by centrifugation and stored at -80°C until assayed. Concentration of E_2 _was determined by a double-antibody RIA using the kit purchased from the Shanghai Institute of Biological Products (Shanghai, China.). The sensitivity of the kit was 1.4 pg/ml, and the intra- and inter-assay coefficients of variation were 3.7–8.0% and 4.74–7.7%.

### Immunohistochemistry analysis

In the Experiment 2, thirty rats divided into the same five groups were used to investigate the expression of GnRH in the rostral medial septum (MS), Broca diagonal band nucleus (DBB) and the medial preoptic area (MPOA) of the rats by immunohistochemistry according to the Atlas [6]. At the days of vaginal opening in the model group, following anesthesia, all of the animals were exsanguinated with normal saline. Perfusion done, the brains were removed and split equally along the third ventricle. One half of the brain was postfixed for >48 h in 4% paraformaldehyde in 0.1 M PB (PH 7.4) with 30% sucrose, and their sections were sliced at 35 μm thickness on a vibratome microslicer and stored at 4°C in tissue culture wells containing 0.1 M PBS (PH 7.4) plus 0.02% sodium azide until further processed. The other half of the brain was snap-frozen in liquid nitrogen, and then stored at -80°C.

Washed in PB for 30 min at room temperature (RT), the floating sections were incubated in PB containing 10% bovine serum albumin (BSA) for 2 h at RT, then with both rabbit anti-GnRH (1:2000, Chemicon International Inc.) diluted in PB containing 1% BSA, 0.02% sodium azide, and 0.4% Triton-X 100 at 37°C for 2 h, and then at 4°C for 70 h. When they had been rinsed in PB (× 3, 5 min each), the sections were incubated in secondary antibody solutions (goat anti-rabbit IgG conjugated to peroxidase 1:200, Sino-American Technology Company, China) with the same blocking sera for 2 h at RT. After that, they were washed (× 3, 30 min), and diaminobenzidine (DAB) was used as chromogen (Vectastain Elite kits, Vector Labs).

### Tissue collection andtotal RNA preparation

The other half of the brain in Experiment 2 was used to investigate the expression of GnRH mRNA by RT-PCR. The target regions, including mediobasal hypothalamus and the suprachiasmatic-preoptic area were dissected (limited anteriorly by the optic chiasma, laterally by the hypothalamic fissures, posteriorly by the mammilary bodies and in depth by the subthalamic sulcus). Total hypothalamic RNA was extracted using 'TRIzol Regent' (Biobasic Inc., Canada) according to the manufacturer's instructions. The purity and integrity of the RNA were checked spectroscopically and by gel electrophoresis before carrying out the analytical procedures.

### RNA analysis

Tissue RNA (2 μg) was reverse transcribed, in a final volume of 20 μl, using 200 IU M-MLV reverse transcriptase in the presence of 25 pmol GnRH specific downstream primer (Sangon Inc), 0.5 mM deoxy-NTP and 20IU Rnasin (from Promega) for 60 min at 42°C, then heat denatured for 5 min at 95°C. 5 μl cDNAs were further amplified by PCR using 25 pmol of upstream primer (Sangon Inc) for GnRH: 5'-ATT CTA CTG ACT TGG TGC GTG-3'; downstream, 5'-GGA ATA TGT GCA ACT TGG TGT-3' [7]. PCR was performed for 30 cycles (1 min at 94°C, 45 sec at 62°C, 1 min at 72°C) in the presence of Taq DNA polymerase (3U per tube) and 2.2 mM magnesium chloride (from Promega) in a final volume of 50 μl. To check the presence of DNA contamination RT-PCR was performed on 2 μg of total RNA without M-MLV reverse transcriptase (negative control). An internal control (β-actin, upstream, 5'-AAG CAG GAG TAT GAC GAG TCC G-3'; downstream, 5'-GCC TTC ATA CAT CTC AAG TTG G-3') for each RT-PCR was performed to account for procedural variations. For each sample 5 μl of PCR amplification products were analyzed on 2% agarose gels and stained with ethidium bromide. Standard DNA (100 bp DNA ladder Promega) was run to provide the appropriate size marker. The RT-PCR products were extracted and purified from agarose gel by Golden Beads Gel Extraction kit (Sangon Inc., China) and sequenced using radioactive dideoxychain terminating method (Sangon Inc., China). The intensities of the bands were evaluated by Image Master Software (SYDR-1990, SYNGENE, U.S.A.).

### Statistical analysis

All data are presented as means ± S.E.M. Statistical analysis was performed on raw data using one-way analysis of variance (ANOVA), with the significance concentrations of p < 0.05 in two-tailed testing chosen. Comparisons among groups were made using the Student's t-test.

## Results

### The day of vaginal opening and regular estrous cycle of the rats

The day of vaginal opening and regular estrous cycle were significantly (p < 0.01, respectively) advanced in M than that in N and V, and there was no difference between N and V groups (Table 1). The day of vaginal opening and regular estrous cycle in HM were significantly (p < 0.01) delayed than those in M and S (Table 1).

**Table 1:** The day of vaginal opening and regular estrous cycle of the rats

	N (n = 18)	V (n = 18)	M (n = 18)	HM (n = 18)	S (n = 18)
Day of vaginal opening	34.00 ± 0.98	34.33 ± 1.09	25.97 ± 2.24*	32.56 ± 1.04^#^	24.78 ± 1.01*
Day of regular estrous cycle	41.26 ± 2.35	45.00 ± 1.19	32.43 ± 0.75*	40.58 ± 0.84^#^	34.56 ± 0.89*
Blood estrogen (pg/ml)	22.64 ± 3.69	24.58 ± 3.87	50.36 ± 5.54*	30.98 ± 7.02^#^	51.14 ± 4.98*

*p < 0.05 vs N and V, respectively; # p < 0.05 vs M and S, respectively

N: intact normal, M: IPP model, V: vehicle with no IPP, HM: IPP model exposed to herb mixture, S: IPP model exposed to saline

### Blood concentration of estrogen

The blood E_2 _concentration in M is twofold higher than that in N (p < 0.05). While the E_2 _level was lower in HM than in S (p < 0.05). There were no differences between N and V, M and S (Table 1).

### Effects of herb mixture on GnRH expression by immunohistochemistry

**Figure 1:**
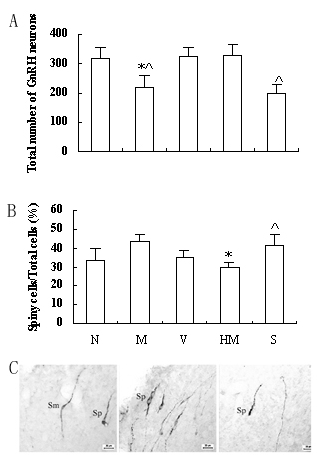
**Effects of herb mixture on GnRH expression by immunohistochemistry. **N: intact normal, M: IPP model, V: vehicle with no IPP, HM: IPP model exposed to herb mixture, S: IPP model exposed to saline. A: The statistical analysis of (spiny GnRH cells/total cells) % (n = 6). * p < 0.05 vs M; ^ P < 0.05 vs HM. B: Total GnRH cells of the rats (n = 6). * p < 0.05 vs N and V, respectively; ^ P < 0.05 vs HM. C: Light micrography illustrating the different types of GnRH cells at the region of MS at low power (n = 6). Sm: smooth type cell; Sp: spiny type cell.

GnRH cells bodies, which were abundant in MS, DBB and MPOA, were calculated. The GnRH cells number in M was less than that in V (p < 0.05), and there was no difference between N and V (Fig 1). The number was significantly higher in HM than that in S (p < 0.05), and no statistical difference was observed between HM and N (Fig 1). There was no significant difference between M and S (Fig 1). The tissue sections processed for immunohistochemistry using antiserum after preabsorption with excessive antigens and omission of primary antibody showed the staining as expected.

The subtypes of all the observed GnRH neurons were discerned under high microscope (× 400). The classification of spiny type and smooth type GnRH neurons was according to the report by Witkin [8] (Fig 1). The percent of spiny type GnRH neurons was significantly higher in M than those in N and HM (p < 0.05, respectively) (Fig 1). There were no differences between N and V, M and S.

### Effects of herb mixture on GnRH mRNA expression by RT-PCR

**Figure 2:**
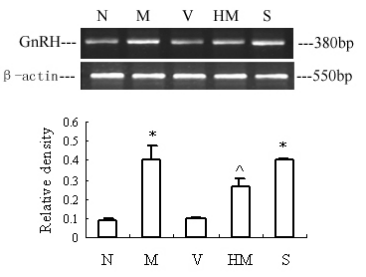
**Effects of herb mixture on GnRH mRNA expression by RT-PCR. **The upper picture shows the gel electrophoresis of the RT-PCR products for the GnRH. Densitometric analysis of the mRNA concentration using GnRH/β-actin expressed as the mean with SEM bar (n = 6) in each column indicated in the lower panel. N: intact normal, M: IPP model, V: vehicle with no IPP, HM: IPP model exposed to herb mixture, S: IPP model exposed to saline. *p < 0.05 vs N and V, respectively; ^ p < 0.05 vs M and S, respectively.

Comparison of the amplified PCR fragment with rat GnRH sequence revealed 100% homology (data not shown). Densitometric analysis of the mRNA concentration using GnRH/β-actin expressed as the mean with SEM. The ratio of GnRH to β-actin in the M increased significantly compared with that in the N and V (p < 0.05), and the ratio in the HM decreased, with no difference showed between HM and N. (Fig 2). There was no significant difference between M and S.

## Discussion

Mammalian sexual maturation and adult reproductive function are centrally controlled by a subset of neuroendocrine neurons that produce GnRH. Recent ten years study indicated, GnRH neurons are the final common pathway for the neuronal control of gonadotropin secretion, which will in turn stimulate gametogenesis and gonadal hormone secretion [9]. In humans and rats, the onset of puberty is highlighted by the augmentation of pulsatile GnRH secretion [10,11]. GnRH release is in a state of low level before puberty, an increased pulsatile release of GnRH is essential for the onset of puberty. True precocious puberty is commonly idiopathicly mediated by premature increasing secretion of GnRH, and is readily treated with potent GnRH agonists [4].

Though GnRH neurons in CNS are paucity, about 600–2,000 cells in mammals, the highest density of the cells is located in MS, DBB and MPOA, approximately 50 to 70% of which project to the median eminence (ME), thus contributing to the control of LH secretion from the adenohypophysis [12]. From this point of view, we selected these regions to observe the change of GnRH expression. Morishito et al has reported the animal model that neonatal treatment with Danazol may induce the true precocious puberty in female rats [3], but the hypothalamic GnRH expression was not examined. In the present study, the high level of GnRH mRNA expression was observed in the M, but the number of the GnRH neuron decreased. The possible mechanism of the inconsistency change might due to the super-release of hypothalamic GnRH in the model rats. Nevertheless, our results, the GnRH mRNA expression showed a higher level in the precocious puberty model rats than in the same age normal rats, the blood estrogen concentration in the model group is twofold higher than that in normal one, along with the day of vaginal opening and the onset of regular estrous cycle advanced significantly in the model rats, give more evidence to sustain the hypothesis that the model induced by neonatal treatment with Danazol is a true precocious puberty model, and might be served to study the onset mechanism of puberty. Besides, though experimental models of precocious puberty have been induced in female rats from 15 to 30 days of age [11,13] or during neonate, by testosterone, estradiol, melatonin and so on, this model appears the subsequent normal ovulatory cycles during adulthood [3].

Danazol, an isoxazol derivative of 17α-ethinylestosterone, is known to have various effects on the reproductive system. Danazol binds to androgen and progesterone receptors, and possesses weak androgenic activity in rats. But the mechanism and the action site of Danazol, administered during the neonatal period, induces a true precocious puberty is not clear. It is generally accepted that the rat hypothalamus is immature at birth and that manipulations of maturation process are possible between days 1 and 10 of life. From these viewpoints, it is possible that the neonatal administration of Danazol may affect the hypothalamic pituitary axis with the rapid rate of maturation, producing a true precocious puberty.

GnRH neurons display a range of shapes from smooth-contoured to extremely irregular or spiny, which reflects or can be modified by the gonadal steroid milieu [8]. The spiny on the GnRH neuron has been identified the synapse by its ultrastructure, suggesting that more synapse connections exist among GnRH neurons and other components in the GnRH network. In the present results, the percent of spiny type GnRH neuron was significantly higher in M than those in N and HM, which might indicate that, on one hand, the gonadal steroid concentration in the local brain region was high; on the other hand, more factors were involved in the process. The local brain aromatization has been observed to be increased in the M [14], which indirectly reflects the increased estrogen concentration. The factors controlling the increased and abnormal secretion of GnRH at the onset of precocious puberty are now being investigated in the same model by us, in which TGF might be an important factor. But further study is carried out. The animals in M and HM groups displayed neural changes to the different degree, suggesting that the herb mixture played certain regulatory effects on the precocious puberty rats.

We have, successfully used the Nourishing "Yin"-Removing "Fire" Chinese Herb Mixture, for the management of IPP for more than thirty years. In 1990', one hundred and six clinical cases have been reported [5]. Before and after treatment, GnRH stimulating test, size of uterus and ovary, X-ray bone age measurement were adopted to estimate the effect of the herb mixture on HPOA and development of internal genitalia and skeleton. Clinical studies indicate that the herb mixture could markedly reduce the blood levels of FSH, LH, E_2 _of the patients, and the volume of uterus and ovary as well. The herb mixture could also inhibit the excessive functional activities of osteoblasts and the decelerated linear growth of skeleton. Besides, the cheap-effective therapeutic approach is more suitable to our national condition than the high-price GnRH agonist.

Though the physiology of human and rodent is not same, the therapeutic effects of the herb mixture on not only true precocious puberty patient but also rat have been observed. The mechanism of the effective drug has been further studied focusing on the neuroendocrine gene expression with modern medical techniques. Recently, seven differential displayed genes between M and HM rats in the same target brain region, have been screened out, by the means of RNA arbitrarily primed PCR (RAP-PCR) (unpublished data). Hopefully, with the all-right therapeutic effect, the herb mixture compound might bring more profits for patients throughout the world.

## Conclusion

The true precocious puberty model by neonatal administration of Danazol in female rats showed augmented expression of hypothalamic GnRH; the Nourishing "Yin"-Removing "Fire" Chinese Herb Mixture down-regulated the increased GnRH expression, and significantly delayed the sexual development of the precocious puberty rat.

## Authors' contributions

Zhanzhuang Tian and Hong Zhao designed the study, performed the animal and molecular genetic studies, and drafted the manuscript. Yan Sun performed the statistical analysis. Depei Cai participated in the coordination. Boying Chen conceived of the study, and participated in its design and coordination. All authors read and approved the final manuscript.
